# Development of a Pandemic Awareness STEM Outreach Curriculum: Utilizing a Computational Thinking Taxonomy Framework

**DOI:** 10.3390/educsci11030109

**Published:** 2021-03-09

**Authors:** Pamela O. Gilchrist, Alonzo B. Alexander, Adrian J. Green, Frieda E. Sanders, Ashley Q. Hooker, David M. Reif

**Affiliations:** 1 The Science House-College of Sciences, North Carolina State University, Raleigh, NC 27604, USA; 2 College of Education, North Carolina State University, Raleigh, NC 27604, USA;; 3 Bioinformatics Research Center, North Carolina State University, Raleigh, NC 27604, USA;; 4 Environmental Health & Safety, Wake Technical Community College, Raleigh, NC 27603, USA;; 5 Chapel Hill-Carrboro City Schools, Chapel Hill, NC 27516, USA;

**Keywords:** science learning, STEM competencies, informal learning, computational thinking, education, curriculum development, data science, teaching, problem solving

## Abstract

Computational thinking is an essential skill in the modern global workforce. The current public health crisis has highlighted the need for students and educators to have a deeper understanding of epidemiology. While existing STEM curricula has addressed these topics in the past, current events present an opportunity for new curricula that can be designed to present epidemiology, the science of public health, as a modern topic for students that embeds the problem-solving and mathematics skills of computational thinking practices authentically. Using the Computational Thinking Taxonomy within the informal education setting of a STEM outreach program, a curriculum was developed to introduce middle school students to epidemiological concepts while developing their problem-solving skills, a subset of their computational thinking and mathematical thinking practices, in a contextually rich environment. The informal education setting at a Research I Institution provides avenues to connect diverse learners to visually engaging computational thinking and data science curricula to understand emerging teaching and learning approaches. This paper documents the theory and design approach used by researchers and practitioners to create a Pandemic Awareness STEM Curriculum and future implications for teaching and learning computational thinking practices through engaging with data science.

## 1. Introduction

Many reports over the last twenty years have emphasized the importance of STEM careers to both the United States and the global workforce [1]. Nowhere has this been more cited than in *A Framework for K-12 Science Education*, which in 2002 articulated eight essential elements of a complete K-12 science and engineering curriculum [2]. Common Core math guidelines from 2010 explicitly call for the use of “technological tools” that aid in the understanding of mathematical concepts [3]. These elements also served as the foundation for the *Next Generation Science Standards* (NGSS) in 2013, where they were situated within core concepts of the fields of science. Many of these practices are familiar to those that teach science regularly, e.g., Developing and Using Models, Planning, and Carrying Out Investigations. The practice of Using Mathematics and Computational Thinking is more complicated. Mathematics as a tool for science is fairly common; however, computational thinking is a less commonly utilized and well-defined practice [2]. Mathematical and computational thinking has been defined by NGSS as:

“Mathematical and computational thinking in [grades] 6–8 [that] builds on K–5 experiences and progresses to identify patterns in large data sets and to use mathematical concepts to support explanations and arguments.

- Use digital tools (e.g., computers) to analyze vast data sets for patterns and trends.
- Use mathematical representations to describe and support scientific conclusions and design solutions.
- Create algorithms (a series of ordered steps) to solve a problem.
- Apply mathematical concepts and processes (e.g., ratio, rate, percent, basic operations, simple algebra) to scientific and engineering questions and problems.
- Use digital tools and mathematical concepts and arguments to test and compare proposed solutions to an engineering design problem.” [4].

Computational thinking and mathematical thinking are intertwined and often confused. Computational thinking is based on computer technology to help collect, visualize and analyze problems. In contrast, mathematical thinking is grounded in developing situational mathematical skills (i.e., geometry and algebra). The *Framework* describes how computational thinking might be employed as the “tools and strategies allow scientists and engineers to collect and analyze large data sets, search for distinctive patterns, and identify relationships and significant features in ways that were previously impossible.” [3]

Unfortunately, these concepts are ill-defined in the standards and often challenging to employ, given the limited understanding of computational thinking outside of a computer science framework. Weintrop et al. [5] outlined a taxonomy that more specifically applied computational thinking concepts and their use as applied explicitly to science and mathematics. Their taxonomy consisted of four categories: data practices, modeling and simulation practices, computational problem-solving practices, and systems thinking practices (p. 128). This work will use that taxonomy and focus on applying data science as a framework to develop the computational thinking skills of students participating in STEM outreach.

## 2. Literature Review

While it may be obvious, access to scientific and mathematical data has never been more available to educators or students than it is currently. Thanks to tools like Google and Wolfram Alpha, data mining and aggregation have become common tools for those in the computer science fields. Concurrently, developing students’ skills in understanding and manipulating this data has also become part of science and mathematics curriculums. For many, this field was called statistics until the 21st century. Not coincidentally, statistics bachelor’s degrees more than doubled between 2008 and 2013 [6]. However, the term data science, as suggested by Cleveland [7], has become the way of describing “the computational aspects of carrying out a complete data analysis, including acquisition, management, and analysis of data [8].” The complex and often quite broad data sets that a data scientist might encounter support the argument for increased computational thinking skills from those that might potentially work with these data. Computational thinking has developed into a critical cross-cutting concept that allows users to develop novel problem-solving skills while implementing solutions in real-world and simulated scenarios [9].

Research has shown that students who work with data in meaningful ways, such as in inquiry-based environments, increase their ability to think computationally; they can visualize, reconstruct, analyze, and synthesize data more coherently. Data science creates a real-world context for data, allowing students to explore concepts such as sampling bias, issues of causation and correlation, the responsible use of information, and contextual knowledge of real-world problems. While most students’ exposure to mathematical concepts begins in early childhood, the development of connections between computational skills and practical science methods is less prevalent.

### Underrepresented Students and Computational Thinking

The National Commission on Excellence in Education recognized the importance of generating new STEM workforce members that specialize in computer science and technology in their 1983 report A Nation at Risk [10]. Since then, investment in technology access has happened in classrooms across the country in both rural and urban spaces [11]. Unfortunately, technology alone has not been the answer to improving student interest in computer-related careers. The lack of new curricular material or pedagogical methods to leverage this technology is apparent. However, it requires changes that are not yet widespread in traditional teacher education and have not been distilled at the student level [12]. Computer science curricula have existed for years in the K-12 space; however, the focus on basic skills and simple programming tasks instead of higher-order computational thinking and problem-solving has served to dampen student interest in the field [13,14]. When students are asked about computer science careers, they conclude that their jobs are uninspiring, abstract, unrelated to real-world issues [15]. More troublingly, many students report they do not even consider careers in computer science because they view the career as too siloed for the average person to pursue [16]. While the lack of qualified STEM workers for the 21st-century economy is an often-cited statistic, it is less well-known that most of these STEM jobs will require training in computational thinking and basic computer science skills [17]. As recently as 2004, the U.S. produced fewer than 33% of applicants deemed necessary to fill these positions [18].

Opportunities exist to improve these numbers; in particular, students from lower socioeconomic backgrounds, rural students, female students, and other students from traditionally underserved racial and ethnic backgrounds could be a growth space for future computer science professionals [19]. Unfortunately, these students’ schools are the most likely to have limited access to quality C.S. or STEM curricula. Additionally, some of these groups (e.g., women and Black students) are actively discouraged from engaging in the type of advanced coursework that prepares students for future C.S. careers [15,20,21]. Concurrently, groups already overrepresented in these fields take advantage of additional resources both from their schools and their families [15]. Implicit biases lead to underserved students being tracked into remedial computer science courses that serve as gatekeepers along the path to future C.S. careers [15]. The authors of *Stuck in the Shallow End* refer to this as “virtual segregation” and note that like any other issue of civil rights, the lack of these underserved groups in computer science must be shown to be explicit, and intentional efforts must be made to overcome it [22].

As mentioned above, these forces lead to a result that is self-evident: only 3% percent of doctoral degrees in computer science went to AALANA (African American, Latinx American, and Native American) people, while 18% were awarded to women [23]. While bachelor’s degrees awarded to African Americans and Latinos are less skewed, they also are proportionally underrepresented [24]. Similarly, women account for only 18% of B.A.s and doctoral degrees in C.S. [25]. The lack of underrepresented students in the critical STEM fields of computer science and other computational fields has been well documented [25]. The lack of exposure to even basic computer science curricula makes exposure to concepts such as computational thinking and the field of data science even less likely. Creating opportunities for these students to develop strong computational thinking skills will require new methods of reaching students and potential new arenas outside the traditional classroom for these investigations to occur. Data science, the manipulation of structured and unstructured data with computer-based techniques, can serve as a real-world application of these skills while offering programming outside of the formal classroom. This method can alleviate the necessity of new teacher training or large expenditures for additional classes at already budget-stricken K-12 schools. Post-secondary institutions with greater resources can serve as a supplement to the standard K-12 curriculum model by offering outreach opportunities for some of these populations of underrepresented students. Additionally, this method of introducing computational thinking can address student perceptions of data science careers in a way that grounds them in real-world applications [26].

Epidemiology, public health science, is an essential medical field. However, retention efforts to epidemiology careers have declined consistently since the 1980s [27]. Developing STEM experiences centered around epidemiology for outreach programs that serve students underrepresented in the medical fields can increase student awareness of these fields. Simultaneously, the field of public health presents an opportunity to develop students’ problem-solving skills in real-world contexts. Creating this epidemiology-based content was the objective of the Imhotep Academy team.

## 3. Methods

**Figure 1.**
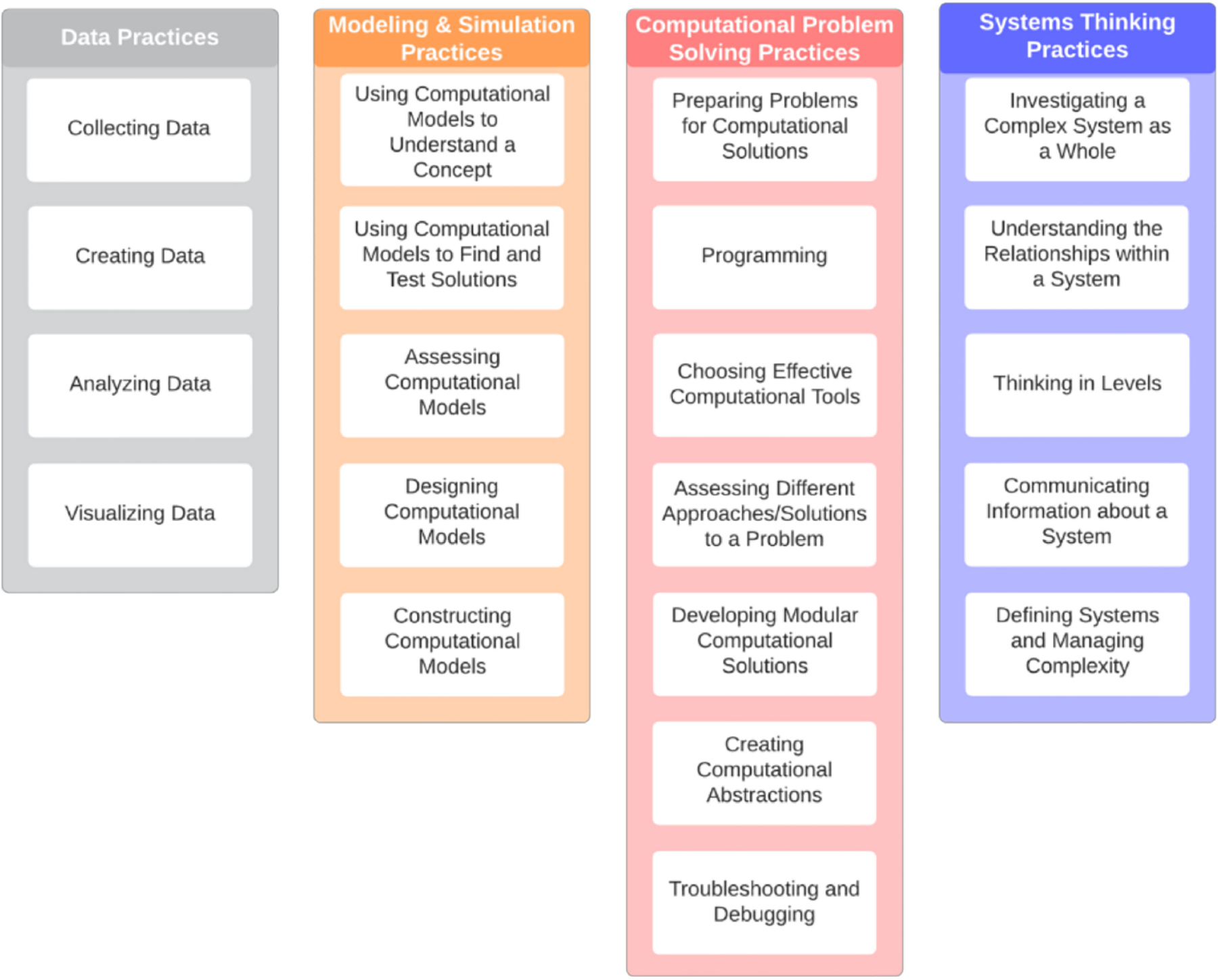
Taxonomy of Computational Thinking from Weintrop, David, Elham Beheshti, Michael Horn, Kai Orton, Kemi Jona, Laura Trouille, and Uri Wilensky, 2016.

Weintrop et al. [5] developed their Computational Thinking in Mathematics and Science Practices Taxonomy to specifically infuse computational thinking into mathematics and science curricula. These four categories are Data Practices, Modeling and Simulation Practices, Computational Problem-Solving Practices, and Systems Thinking Practices. The full taxonomy is shown in Figure 1.

This work aims to show how data science, as a science and mathematical teaching tool, can address multiple aspects of this taxonomy. A data science-based STEM curriculum creates opportunities within each category of the computational thinking taxonomy to develop student practices in mathematics presented in this paper.

### 3.1. Imhotep Academy Program Design

Imhotep Academy Photonics Pre-College Program model (3PM) addresses how to prepare underrepresented minority K-12 students for science, technology, engineering, and mathematics (STEM) careers and to equip teachers and parents with resources to engage learners in these disciplines [28]. The program model is grounded in six components (1) Student Recruitment and Retention; (2) STEM Content; (3) Parental Engagement; (4) STEM Professionals; (5) Leadership and Professional Development; and (6) Evaluation and Dissemination. The bidirectional model focuses on strategies that place student learning and experiences in STEM as the central focus.

The program model is guided by four principles:

1. Immersion in traditional and nontraditional hands-on, problem-based investigation;
2. Engagement in a supportive, safe and challenging environment and support system;
3. Participation in leadership and professional development training;
4. Integration of professionals from academia, industry, and schools with parents and students in synergistic STEM activities, internships, and mentoring relationships.

Each principle is operationalized through one or more of the program components. Immersion in these components enhances participant (i.e., students and teachers) awareness of STEM careers by providing opportunities to participate in authentic learning experiences that expand the awareness of the interdisciplinary connections of STEM while building the 21st-century learning skills participants need for the global workforce.

Over a 20-h intervention, Imhotep Academy engages students in a STEM topic using an interdisciplinary approach to guide students into practicing the skills and behaviors of a scientist, mathematician, engineer, and technologist. Students participate in inquiry-based activities, career exploration activities, mathematics problem-solving scenarios, and interactions with scientists through laboratory investigations and tours. These experiences are used to deepen students’ awareness of STEM disciplines, its rigor, and requirements. Students also research and discuss the contributions of diverse STEM researchers. Mathematics is taught in an applied manner to support the delivery of science content and develop students’ problem-solving skills to interrogate the world through the science context.

Additionally, technology is taught to enhance students’ knowledge of science content through STEM career activities, simulations, and presentations. Students rotate through three classes modeling the role of STEM professionals to solve problems related to grand challenges globally and present their findings at the closure of the program to their parents and peers. The public health crisis provides young learners with an opportunity to discover the intersection of science, mathematics, and problem-solving in a real and hypothetical manner.

### 3.2. Pandemic Awareness Outreach Curriculum Intersection with Computational Thinking and Data Science

The Pandemic Awareness Outreach curriculum aligns with Imhotep Academy’s goal to develop students’ STEM awareness, knowledge, and skills needed to thrive in the global workforce. Imhotep Academy provides the contextual setting for students to learn the mathematics and science underpinning epidemiology and other disease-related fields. In particular, mathematics serves as a tool to make sense of disease spread and impact while providing students with a method to analyze real-world problems and test solutions.

To provide guidance for professional educators, in 2000 the National Council of Mathematics (NCTM) outlined six mathematical practices essential for preparing American students for college and career in the Common Core Standards for Mathematics. These practices include (a) making sense of the world and persevering, (b) reasoning abstractly and quantitatively, (c) constructing viable arguments and critique the reasoning of others, (d) modeling with mathematics, (e) using appropriate tools strategically, and (f) attending to precision [29]. Teaching and learning should be guided by these practices to center K-8 learning within an environment that supports students’ mathematical thinking skills. Sneider, Stephenson, Schafer, and Flick [30] defined the differences between mathematical and computational thinking in how students approach new situations. Students develop mathematics thinking when they approach problems with mathematical skills at their disposal and computational thinking when they approach a new situation with an awareness of how computers can visualize systems and solve problems. In comparing these cognitive skills, researchers noted common competencies of problem-solving, modeling, analyzing, and interpreting data and statistics and probability in both mathematics and computational thinking [30].

**Figure 2.**
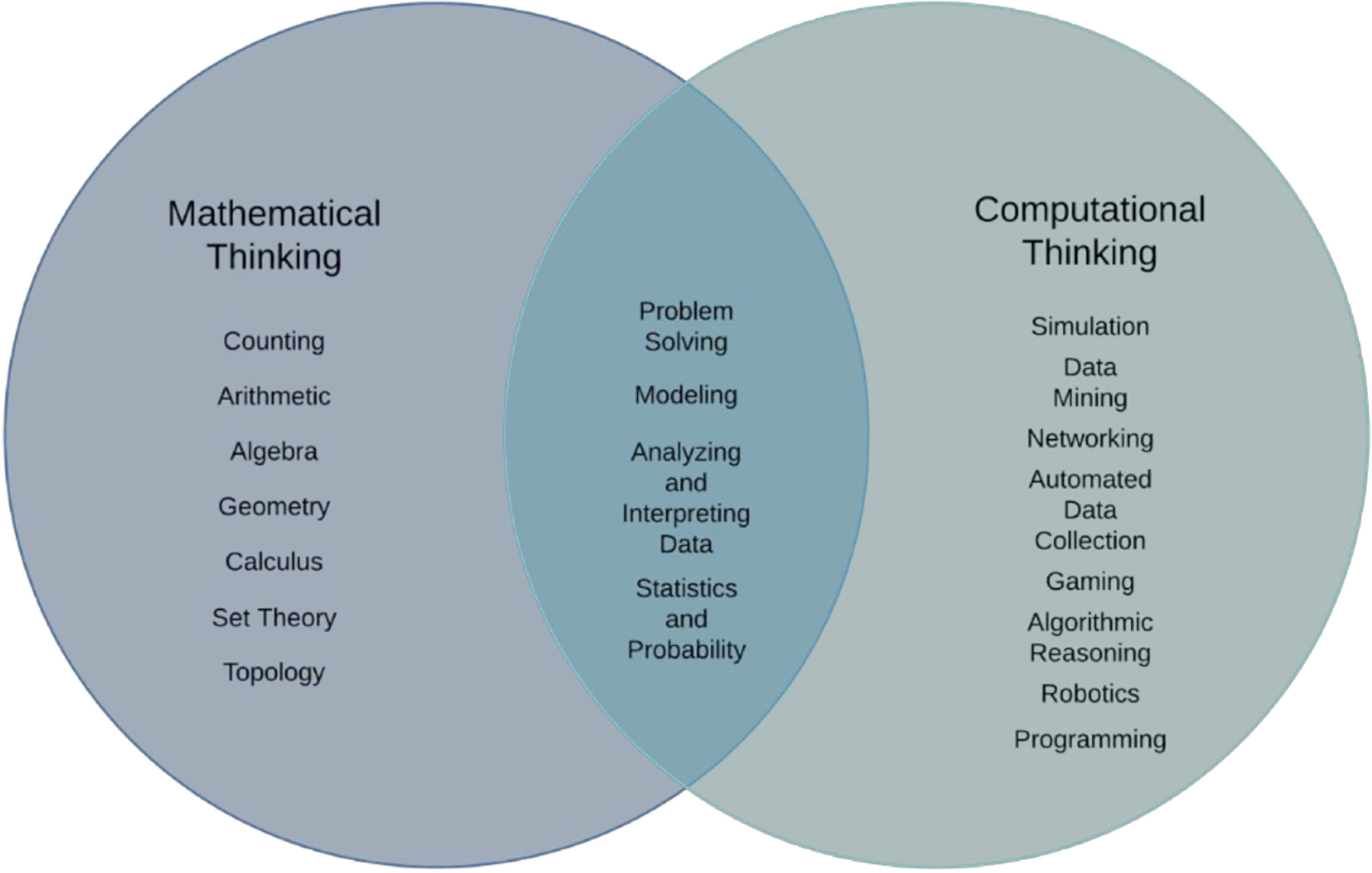
Venn diagram of mathematical and computational thinking. From Sneider, C., Stephenson, C., Schafer, B., and Flick, L., 2014.

These practices guide the development of the requisite skill sets necessary when designing relevant STEM learning experiences and programs for diverse learners. Hence, the development of the Pandemic Awareness STEM Program Outreach Curriculum was grounded on the computational thinking skills taxonomy designed by Weintrop et al. [5] and aligned with the four categories noted by Sneider et al.: problem-solving, modeling, analyzing and interpreting data and statistics and probability [30]. Figure 2 outlines the similarities and differences between mathematical thinking and computational thinking. Mathematical thinking addresses processes like counting arithmetic, algebra, geometry, calculus, set theory, and topology. In contrast, computational thinking includes simulation, data mining, networking, automated data collection, gaming, algorithmic reasoning, robotics, and programming.

Imhotep Academy is a fee-based outreach program at N.C. State University with the mission to engage students traditionally underserved in science, technology, engineering, and mathematics (African American, Latinx, Native American, and girls). The program provides a space to introduce students to the grand challenges investigated within the College of Sciences’ six departments: biological sciences, physics, chemistry, statistics, mathematics, and marine, earth, and atmospheric sciences departments through education programs. The overall goal is to increase the number of students matriculating at four-year universities, pursuing STEM degrees, and preparing for careers in a high-tech world. Annually, Imhotep Academy reaches approximately 200 students from 10 rural and urban counties in North Carolina, 50% female, and 70% African American students. The program is managed by a full-time program director and temporary staff of certified public-school teacher and STEM undergraduate and graduate students serving as instructors. Imhotep Academy partners with STEM researchers within the College of Sciences and extended N.C. State University research community to introduce students to interdisciplinary research applications through problem-based STEM curriculum and experiential learning activities. For this specific session, the program met weekly on Saturdays for three consecutive online hours, where students rotated through three 55-min sessions of science, mathematics, and technology.

### 3.3. Curriculum Development Process Program Design

Imhotep Academy teaching staff and team develop integrated lesson plans and activities aligned with the Next Generational Science Standards (NGSS) and North Carolina Essential Science Standards (NCES) and North Carolina Common Core Mathematics Standards (NCCCMS). All the lessons offered at Imhotep Academy use a pedagogical approach of inquiry and problem-based learning, often with handouts in a face-to-face setting. Typically, the program is offered over a week on the campus of N.C. State. However, given current events, this program session was offered entirely online. The 5E lesson planning tool is used to direct lesson planning, implementation, and evaluation. Program activities are designed using common materials (i.e., paper, cloths, tissue, confectioner’s sugar) found in local stores or an average middle school classroom and then extended by researchers in the field partnering with the program.

#### The Lesson Planning and Proposed Implementation

The lesson planning component consisted of four phases. Each phase focused on developing a curriculum that would engage students in problem-based activities to increase students’ awareness of pathogens, disease transmissions, and the field of epidemiology. These meetings were collaborative, including all voices and the diverse expertise of the team. Each team member was tasked to design a problem-based unit guided by state and national standards in four lessons.

The first phase focused on STEM content selection. The Imhotep PAOC team consisted of a biologist teaching science, a toxicologist with a computer science background teaching mathematics, an elementary science educator teaching the epidemiology career and technology content, a STEM education graduate student with a physics background, and a program director with a middle school STEM education background. During the planning meeting, the team shared potential activities to develop an integrated problem-based curriculum related to the current public health crisis. The science teachers shared activities that introduced pathogens, the epidemiology field, and sample activities related to disease prevention and diagnosis. The team discussed potential alignments and spent a week working on a curriculum outline for four 55-min problem-based activities based on the theme.

During the second phase of the curriculum design, the PAOC team provided a curriculum outline for all three subject areas. The discussion focused on the specific objectives of proposed activities, activity and theme alignment, and activity scope. The science instructor proposed content and activities that would introduce students to pathogens, disease transmission, case analysis, and disease prevention. The mathematics instructor proposed content routed in data science and programming as tools used in the epidemiology field. The technology instructor designed activities that further enhanced the students’ understanding by exposing them to epidemiology specific careers and introducing problem-solving techniques applicable to the discipline.

**Figure 3.**
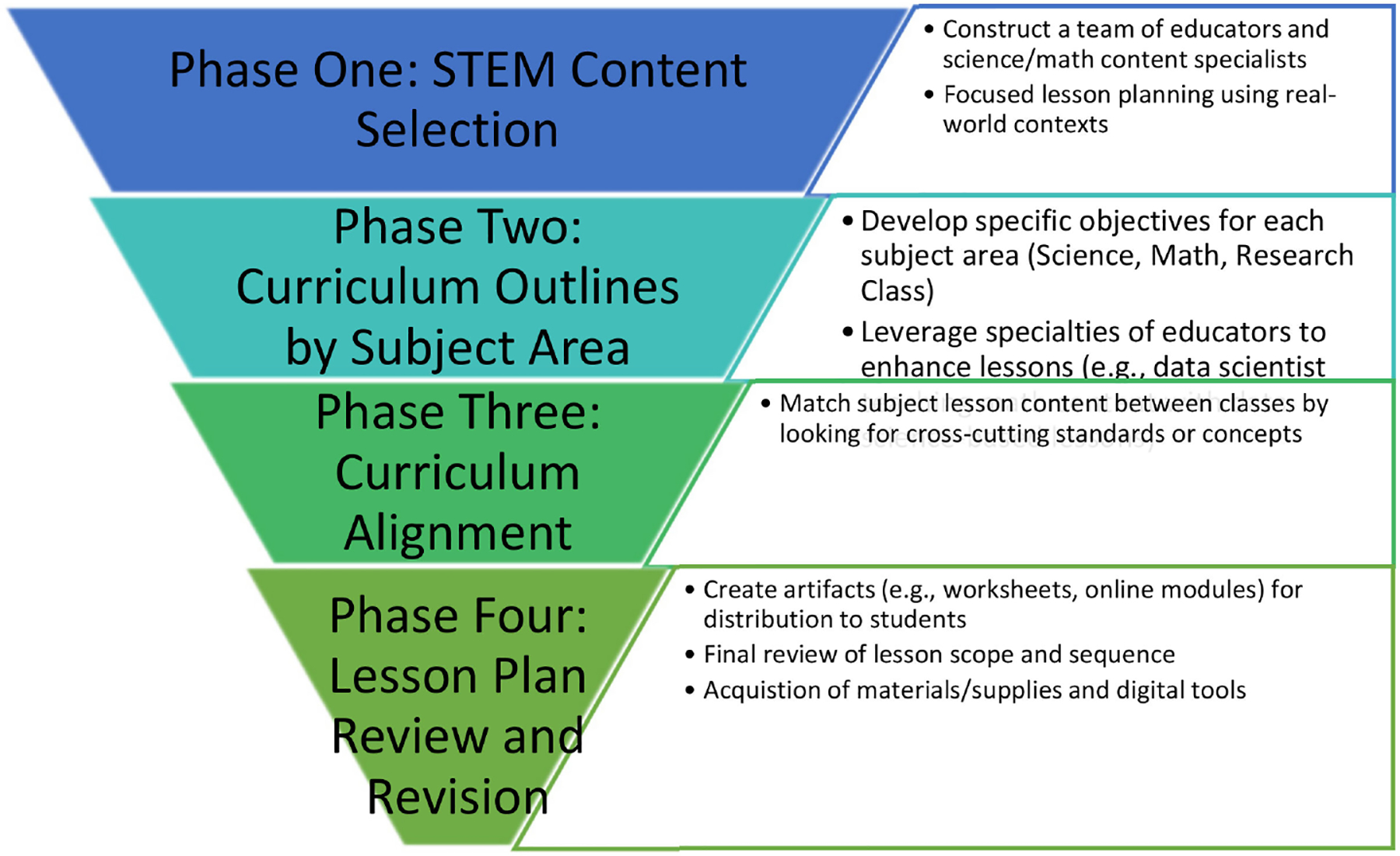
Overview of Lesson Planning Phases.

In the third phase of the curriculum design, individual meetings were held with the content specialists to answer any scope and sequence questions related to science, mathematics, technology data science content, computational tools and a discussion about the delivery of programming instruction before the activities and lesson plans were developed for review. The fourth phase consisted of the PAOC content experts developing draft lesson plans, activities, and a series of content-related assessment questions appropriate for middle school students. These lessons were reviewed again by the program director and STEM Education graduate student to consider the activities’ content, sequence, and scope. The plans and activities were annotated to provide feedback regarding revisions to support optimal instructional implementation. The fourth phase also incorporated a review of the revised sample activities and lessons. It allowed instructors to test their lesson with the PAOC team for feedback prior to delivery with students to support preparation for delivering the content to middle school students. Each phase is summarized in Figure 3. The final result was a full curriculum unit of 12 problem-based activities, an assessment, and guiding questions for final presentations prepared for implementation with middle school students.

## 4. Implementation

The Pandemic Awareness STEM Outreach Program Curriculum goal is to introduce students to epidemiology, the work of epidemiologists, and the requisite knowledge and skills needed for an epidemiologist specializing in disease transmission, treatment, and prevention. Guided by the Next Generation Science Standards, North Carolina Essential Science Standards, Common Core Standards, and International Society of Technology Education Standards, the curriculum integrates science, mathematics, and technologies into a unit consisting of 465 min of problem-based scenarios, simulations, mathematical modeling and simulation activities that guide students to developing a deeper awareness of science, STEM careers, mathematical and computational thinking skills.

### 4.1. Science

In science, students complete hands-on investigations as ‘disease detectives’ in their exploration of pathogens and the aspects of human health in four activities: Virus Explorer Activity and Mask Material Filtrations Experiment, Bacterial Meningitis Outbreak Parts I and II, Patient Zero Handwashing Experiment, and Vaccine Development. Table 1 below describes each science activity.

In a virtual environment, the students will explore the CDC’s Junior Disease Detectives “Operation: Outbreak” digital comic book [28]. The comic is used to investigate case studies and determine causation for a fictional outbreak using the 5 Ws of epidemiology. In the process, the students are exposed to careers in Epidemiology throughout the topic discussion.

### 4.2. Mathematics

In mathematics, students will complete the four activities that develop skills similar to those used by professional epidemiologists: Exploring Pathogen Sizes and Facemask Comparison, Averages and Reproduction Rates in Outbreak Simulators, and Developing Your Own Outbreak Simulator Parts 1 and 2. Table 2 describes these activities.

### 4.3. Technology

In technology, students will complete the following activities to learn about the epidemiology field: What is Epidemiology, Solve the Case, Epidemiology Career Research and Career Research presentation. Table 3 below describes each technology activity.

## 5. Discussion

The Computational Thinking Taxonomy by Weintrop et al. [5] provided a guide for identifying how data science competencies can be taught to engage students in the use of problem-solving techniques in a STEM outreach context. While some activities may only address one computational thinking practice, others naturally encompassed several practices. These activities were designed by the PAOC team to provide learners progressive points of engagement with the content and critical data science skills used by professionals in epidemiology. A similar approach was implemented by Behesti and others in a study covering a variety of STEM disciplines in a formal school setting [31] In their work, STEM doctoral students developed lesson plans in conjunction with high school teachers that incorporated the firsthand experiences of the STEM students into the practices they wanted the high school students to develop. As an essential part of this strategy, all activities will be implemented to provide immersive learning opportunities. For example, the *Developing the Outbreak Simulator* activity encompasses aspects from each of Weintrop’s computational thinking categories: 1. Data Practices, 2. Simulations and Models, 3. Computational Problem Solving, and 4. Thinking in Levels in mathematics and science. The implementation of the PAOC activities over the course of multiple weeks is designed to increase the likelihood of gain in CT skills over time, as seen in work from Jaaipal-Jamani and Angeli [32], who saw increases in CT skills from interventions designed to teach robotics concepts. An explanation of several aspects of this lesson and its specific relationship to the taxonomy is provided below.

The PAOC mathematics unit addressed Data Practices by incorporating activities for students to collect, create, manipulate, analyze, and visualize data related to infectious pathogens. Students will analyze published data on pore sizes of mask materials and simulated disease transmission data to calculate the empirical basic reproduction numbers of sample pathogens using averaging, addition, and multiplication. Learners will later input these data into an internet-based coding platform, Cocalc.com, and visualize how these variables influence the spread of a disease through a viral population.

Modeling and Simulation Practices were addressed in activities designed to engage students in using and constructing computational models to better understand science content related to epidemiology. The activity will develop students understanding of disease transmission and how preventive factors (immunity, medication, and quarantine) might impact disease spread through a computational model. Another aspect of the activity guides students to learn the computational logic needed to build their outbreak simulator using Python code. Students will complete tasks that allow them to manipulate input variables, basic code framework, and run simulations. The learners will use several pathogen-related variables (e.g., red logic models to aid their construction of an outbreak simulator using pseudocode of several variables (reproduction rate, medication, and quarantine) to see the influence of the variables on disease spread in a simulated population. The activity and tasks provide a foundation for students to learn the basic terminology of disease transmission and mitigation and a rudimentary understanding of basic programming statements.

The PAOC addressed the Computational Problem-Solving Practices in activities that expose students to programming through computational logic, Python, and syntax. The participants will complete tasks that require them to add useful functionality to an outbreak simulator using uncustomized, pre-written code blocks. These activities will guide students by manipulating, running, and debugging code to provide a foundational understanding of how logic is essential in computational thinking.

Systems Thinking Practices concentrated on including activities that will guide students to thinking about models as nested levels during the development of a deeper understanding of disease progression and how and when an intervention is most effective. The simulator provides a macro and micro perspective of what happens to an individual during a disease outbreak and their relationship to a larger population. Through discussions, students will be guided to varying quarantine measures into their simulators and discuss their efforts’ effectiveness. The simulator will provide a visual representation of quarantine’s effectiveness for the entire population when differing amounts of individuals follow quarantine guidelines and evaluate how these differences impact disease spread in a population. After completing their outbreak simulator, students will be able to articulate appropriate quarantine measures to stop the spread of different types of infectious types of diseases in a variety of populations.

In the study by Behesti and others, each of these aforementioned categories of the taxonomy were cited by the STEM practitioners as being relevant to CT practices for working in their field of research [31]. Researchers have also noted that CT practices as seen by both STEM professionals and students as overlapping with scientific and mathematical practices in ways that make them effective practice for K-12 students [33,34].

### Future Research and Practice Implications

The next steps will include implementing the Pandemic Awareness STEM Outreach Curriculum to diverse middle school students from urban and rural counties in North Carolina with a significant percentage of historically underrepresented student groups and documenting the teacher implementation and student learning experiences. An examination of underrepresented students participating in STEM learning designed to increase CT skills is especially relevant, as previous studies have been inconclusive in examining whether CT skills are as readily transferred to students with lower starting levels of CT (typical of students with less exposure to quality STEM experiences) compared to their peers [35].

Potential findings from the implementation of the curriculum, reflections of instructors, and survey data from participants in Imhotep Academy, an informal STEM outreach program, may provide useful information on:

1. The actual scope, sequence, and rigor of the data science curriculum activities;
2. How to effectively teach computational and computer science-based curriculum within an integrated STEM context;
3. How students’ attitudes, perceptions, and knowledge are influenced by computational thinking and data science learning experience;
4. How informal settings may serve as a learning laboratory for designing novel or nonexistent computational and computer science curriculum for K-8 schools and teacher professional developments.
5. How to make data science an integrated visual component in solving real-world problems within a STEM context and the world;
6. How to support learners and educators in understanding the theoretical concepts presented practically and engagingly to develop logical reasoning and coding skills;
7. How to increase the interests of AALANA students who are historically underrepresented in the computer science, computational thinking, and STEM fields.

These findings can be used for further investigation by computer scientists, educational researchers, practitioners, and stakeholders to address the need for computer science and computational thinking curriculum in public schools. These efforts should be directed at developing every students’ knowledge of computer science with explicit goals to broaden the participation of traditionally underrepresented and underserved groups of students.

In addition to the impact on students, program educators may also gain teaching and learning knowledge from considering how the taxonomy’s computational and mathematical practices can apply to STEM content being introduced and consider its integration into future lessons they may develop.

## Figures and Tables

**Table 1.** Science Activities.

Day 1: Virus Explorer Activity and Mask Material Filtrations Experiment	Students will explore 3D images of viruses (including coronavirus), examine their shapes, and investigate how they can infect a host cell. Students will determine the effectiveness of five mask materials to learn more about prevention. Students will create, visualize, collect, manipulate, and analyze pathogen data and test their hypotheses.
Days 2 and 3 Bacterial Meningitis Outbreak Parts I and II	Students will investigate an outbreak in a high school and diagnose patient illness, including a protein, glucose, and antibody investigation.
Day 4: Patient Zero Handwashing Experiment and Vaccine Development	*Students will* investigate hand washing techniques and how handwashing can change environmental conditions and vaccine development.

**Table 2.** Mathematics Activities.

Day 1: Exploration of Pathogens Sizes and Facemask Comparison Activity	Students will investigate pathogens and how the pore sizes of common face mask materials influence their effectiveness. The students will work with the metric system and the Biointeractive Virus Explorer to convert the length of viruses and bacteria from nm to μm and then rank them based on size. They will explore the pore sizes of different mask fabrics, use the metric system to convert the pore sizes to μm (from cm, mm, and nm), rank the mask materials by pore size, and then evaluate which type of mask would be the most effective at filtering various pathogens based on size.
Day 2: Averages of Reproduction Rate activity and Outbreak Simulator	Students will use basic statistics to calculate disease reproduction rates (R0) using averages and through the hands-on application of these values using an outbreak simulator.
Days 3 and 4: Develop Your Own Outbreak Simulator Part I and II Activity	Students will be introduced to basic coding IF, WHILE and FOR statements using pseudocode followed by a line-by-line conversion of the pseudocode to Python script. Students will use knowledge from the outbreak simulator and develop coding skills needed to write their own outbreak simulator. They will be introduced to and use a collaborative coding platform, Cocalc, to develop their visual outbreak simulator incorporating R0, incubation time, infectious duration, immunity, medical treatment, and quarantine measure to simulate the spread of a disease through a population.

**Table 3.** Technology Activities.

Day 1: What is epidemiology, and what is an epidemiologist?	Students will be introduced to the work and role of an epidemiologist and the broader field of epidemiology. Students complete an Epidemiologist simulation to calculate odds ratios and solve mathematical problems related to solving the spread of disease.
Day 2: Solve the Outbreak	Students will apply the problem-solving technique to solving an outbreak case.
Day 3: Epidemiology Career Research	The students will pick one of the following careers: Pharmaceutical epidemiologist; Infection control epidemiologist; Medical epidemiologist; Infection disease epidemiologist; Field epidemiologist; Molecular epidemiologist; or Veterinary epidemiologist. Next, they will locate pertinent information regarding the career role, education requirements, salary, etc.
Day 4: Career Research Presentation	Students will present their career research to their peers.
